# Unmasking Invasive Pulmonary Aspergillosis: Insights From a Case Series at a Tertiary Care Center

**DOI:** 10.7759/cureus.100065

**Published:** 2025-12-25

**Authors:** KVP Munasinghe, AM Nanayakkara, WDN De Zoysa, D Rodrigo, GBL Samarasekera

**Affiliations:** 1 Respiratory Medicine, National Hospital for Respiratory Diseases, Welisara, LKA; 2 Venereology, National STD/AIDS Control Programme, Ministry of Health, Colombo, LKA

**Keywords:** amphotericin, diabetes mellitus, hydropneumothorax, invasive pulmonary aspergillosis, voriconazole

## Abstract

Invasive pulmonary aspergillosis (IPA) is a life-threatening fungal infection that primarily affects individuals with significant immunosuppression, including those receiving treatment for hematological malignancies or undergoing solid organ or hematopoietic stem cell transplantation. Additional risk factors include severe or prolonged viral infections such as influenza and COVID-19 requiring intensive care, as well as underlying conditions such as diabetes mellitus and chronic granulomatous disease. Although the clinical course could be variable, the initial presentation is often ill-defined. All three patients in this case series presented with fever and productive cough, and one developed hemoptysis during the ward stay. Diagnosis was established using a combination of radiological imaging, bronchoscopy with bronchoalveolar lavage for fungal cultures, and galactomannan antigen testing. Radiological findings were diverse, ranging from tree-in-bud nodules and cavitating consolidations to the rare occurrence of hydropneumothorax. Despite the importance of early recognition and prompt antifungal therapy, diagnosis and management remain challenging. This case series highlights the heterogeneous manifestations of invasive pulmonary aspergillosis, the diagnostic and therapeutic difficulties encountered, and the complexities of antifungal selection in the presence of organ dysfunction. Early detection and a multidisciplinary approach are essential for improving clinical outcomes. In situations where microbiological confirmation is not feasible, a high degree of clinical suspicion, characteristic radiological findings, and a favorable clinical response to empirical antifungal therapy may improve clinical outcomes.

## Introduction

Invasive pulmonary aspergillosis (IPA) is an infection characterized by the invasion of the lung tissue, often with angioinvasion by *Aspergillus* species, leading to systemic dissemination and tissue necrosis [1]. *Aspergillus fumigatus*, *Aspergillus flavus*, *Aspergillus niger*,and *Aspergillus* *terreus *are some of the causative agents, among which *Aspergillus fumigatus *acts as the commonest [2]. Global estimates of invasive aspergillosis incidence range around 250,000 cases per year. Patients with profound immunosuppression, including those receiving therapy for hematological malignancies or undergoing solid organ or hematopoietic stem cell transplantation, are at high risk for invasive fungal infections. Additional risk factors include severe or prolonged viral infections, such as influenza and COVID-19, requiring intensive care, as well as underlying conditions such as diabetes mellitus and chronic granulomatous disease, all of which compromise host defenses and predispose patients to invasive fungal disease [3]. Managing invasive pulmonary aspergillosis, particularly in a patient with multiple comorbidities, is intricate, as selecting the appropriate antifungal agent and ensuring its safe continuation require close surveillance.

In this case series, we present three patients with microbiologically confirmed pulmonary aspergillosis who presented to a leading tertiary care respiratory center in Sri Lanka, highlighting the multifaceted clinical spectrum, the diagnostic and therapeutic challenges encountered, and the outcomes while emphasizing the importance of early recognition and aggressive treatment.

## Case presentation

Case 1

A 76-year-old man, a known patient with diabetes mellitus with erratic glycemic control, hypertension, and ischemic heart disease, presented with an intermittent fever and a productive cough with yellowish sputum for a duration of two weeks. There were no hemoptysis or significant weight loss and no history of or known contact with tuberculosis (TB).

On examination, he was febrile but hemodynamically stable, with an oxygen saturation (SpO₂) of 98% on room air, a blood pressure of 130/80 mmHg, and a pulse rate of 110 beats per minute (bpm). He was not in respiratory distress. Lung auscultation revealed coarse crepitations scattered throughout both lung fields.

**Figure 1:**
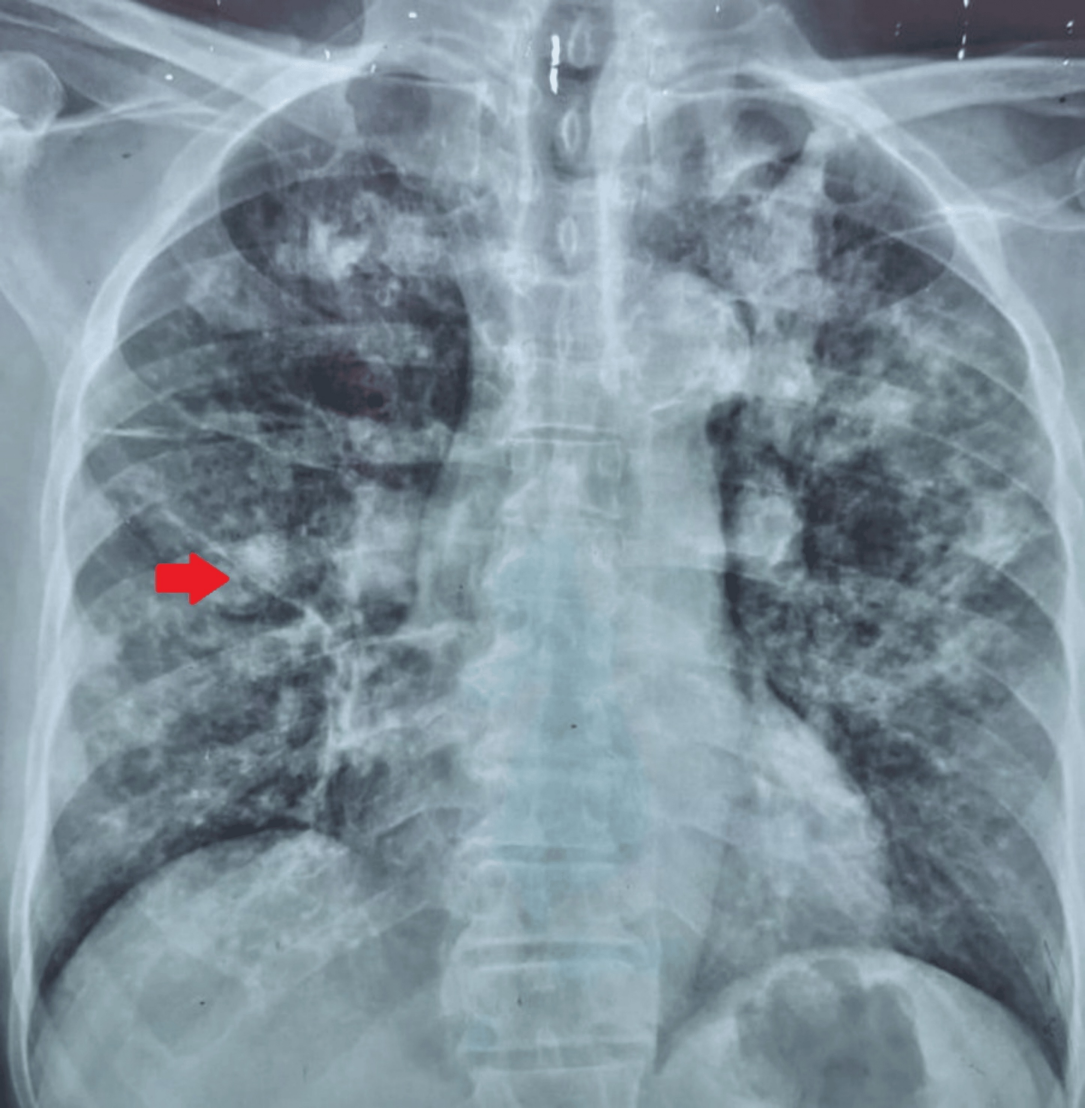
Chest radiograph (patient 1) Chest radiograph showing bilateral patchy consolidations with nodularity. The red arrow indicates a nodule in the right middle lobe close to the right hilum

His hematological investigations showed neutrophilic leukocytosis with markedly elevated inflammatory markers. Chest radiography (Figure 1) demonstrated bilateral inflammatory shadows with nodularity. Sputum culture grew coliform species, but the sputum for liquid culture was negative for TB. Sputum galactomannan was positive (Table 1).

**Table 1:** Investigations and reference values

Test	Reference range	Case 1	Case 2	Case 3
White blood cells (×10^9^/L)	4.0-11.0	18	16	19
Neutrophils (%)	40-75	70	68	72
Hemoglobin (mg/dl)	12.0-16.0	11	8	8.9
Platelets (×10^9^/L)	150-450	420	180	437
C-reactive protein (mg/dL)	<6	340	288	242
Erythrocyte sedimentation rate (mm/hour)	<20	98	110	115
Serum sodium (mmol/L)	135-145	133	130	131
Serum potassium (mmol/L)	3.5-5.5	3.3	3.2	4.3
Serum creatinine (mg/dL)	0.7-1.3	1.6	2.0	3.1
Aspartate aminotransferase (U/L)	8-33	160	135	149
Alanine aminotransferase (U/L)	4-36	145	170	153
Serum bilirubin (mg/dL)	0.2-1.3	1.8	2.0	1.3
Fasting blood sugar (mg/dL)	<110	308	280	178
Serum galactomannan	<0.5	2.02	-	-
Bronchoalveolar lavage (BAL) galactomannan	<0.5	7.92	-	2.41

**Figure 2:**
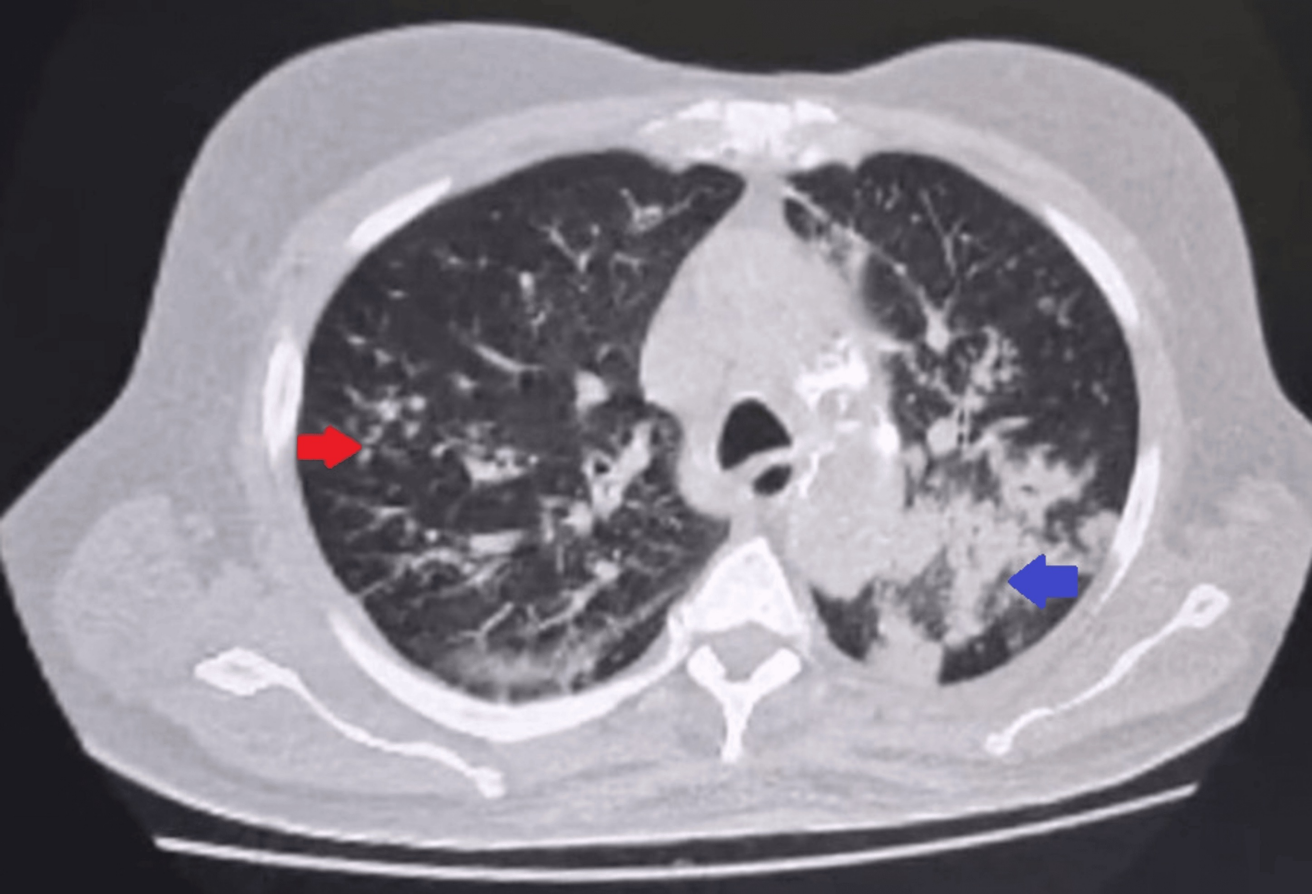
High-resolution computed tomography (HRCT) of the chest: axial view (patient 1) HRCT of the chest illustrating multiple tree-in-bud nodules in both lung fields (the right arrow indicates tree-in-bud nodules in the right upper lobe posterior segment) with peribronchial consolidations bilaterally (the blue arrow indicates peribronchial consolidations in the left lower lobe superior segment)

On admission, he was started on intravenous ceftriaxone 1 g twice a day (bd) and oral clarithromycin 500 mg bd. However, as there was minimal clinical improvement after 48 hours, antibiotics were escalated to intravenous meropenem 1000 mg bd, a renal-adjusted dosage after discussion with the microbiology team, which was continued for 14 days, though the response remained suboptimal. High-resolution computed tomography (HRCT) of the chest (Figure 2) revealed multiple tree-in-bud nodules in both lung fields with peribronchial consolidations bilaterally, including the superior segments of both lower lobes, in keeping with invasive fungal pneumonia.

Bronchoscopy was performed, and bronchoalveolar lavage (BAL) galactomannan returned highly positive (7.92), while fungal culture isolated* Aspergillus fumigatus*. BAL for TB was negative.

As he initially had elevated transaminases and impaired renal function, he was commenced on intravenous liposomal amphotericin 200 mg daily for 10 days after discussion with the mycology team. Therapy continued until liver transaminases normalized, after which he was transitioned to oral voriconazole 200 mg bd.

He showed remarkable improvement with antifungal therapy, and his fever completely resolved.

Case 2

A 43-year-old man with diabetes and poor glycemic control presented with an intermittent fever, constitutional symptoms, and a productive cough for a duration of three weeks. He did not complain of hemoptysis on admission, and there was no known contact history of TB.

On examination, he was febrile, and lung auscultation revealed coarse crepitations with patches of bronchial breathing over the left upper zone. Blood investigations showed neutrophilic leukocytosis with markedly elevated inflammatory markers. Sputum screening for pyogenic organisms, TB, and fungi was negative.

After discussion with the microbiology team, empirical broad-spectrum antibiotic (piperacillin-tazobactam) 4.5 g every eight hours was commenced and continued for 10 days, but there was minimal clinical improvement. Given the clinical suspicion of tuberculosis, the patient was initiated on a weight-based, drug-sensitive antituberculosis regimen consisting of a fixed-dose combination of isoniazid, rifampicin, ethambutol, and pyrazinamide for the two-month intensive phase. Bronchoscopy was arranged, and bronchoalveolar lavage samples were obtained for TB and fungal studies.

**Figure 3:**
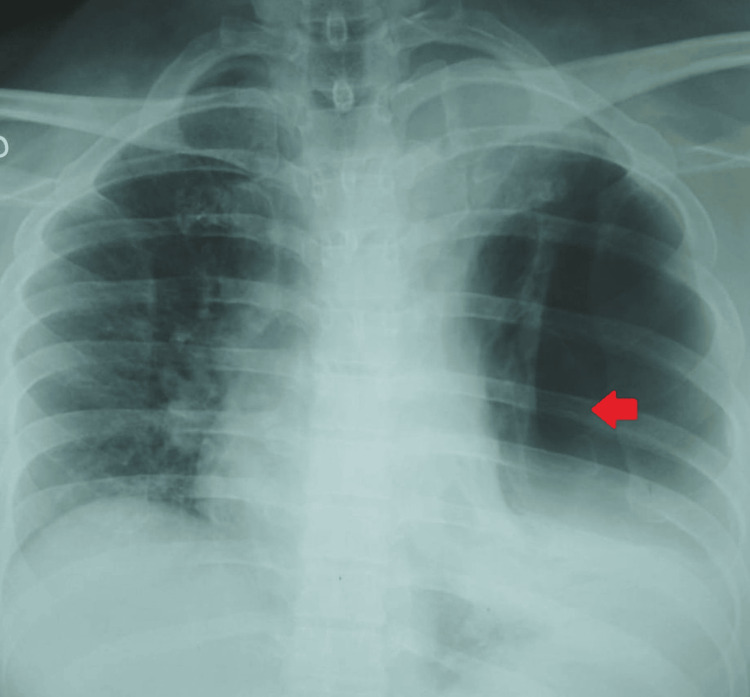
Chest radiograph (patient 2) Chest radiograph illustrating left-sided hydropneumothorax with mediastinal shift to the right side. The red arrow indicates the left-sided pneumothorax with a collapsed lung margin

**Figure 4:**
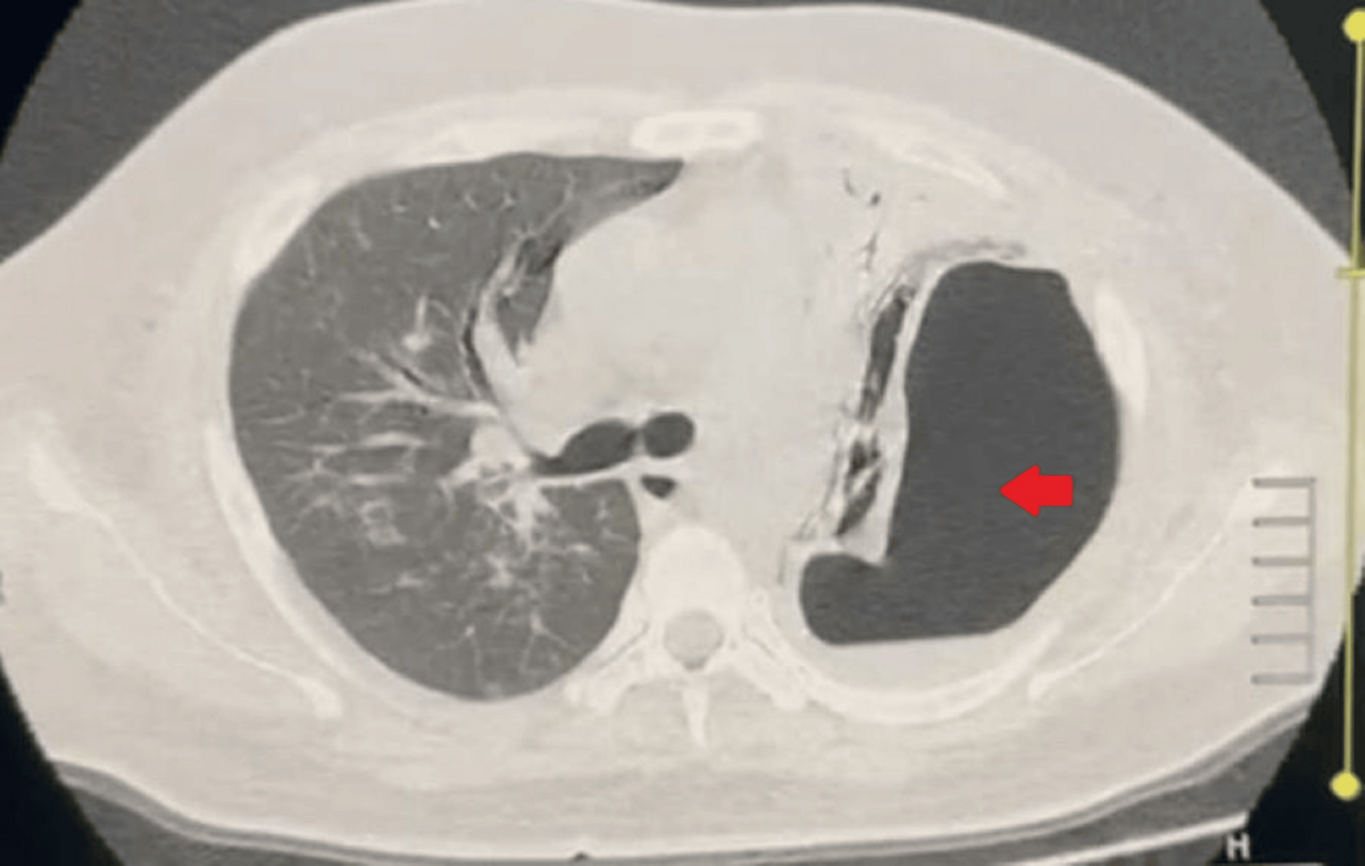
HRCT of the chest: axial view (patient 2) HRCT of the chest demonstrating an encysted left-sided hydropneumothorax measuring 10 cm × 7.8 cm × 12 cm with collapsed left lung. Peribronchovascular patchy consolidation with ground glassing noted in the right lung with upper lobe predominance. The red arrow indicates the left-sided pneumothorax HRCT: high-resolution computed tomography

During his hospital stay, he developed acute-onset shortness of breath, followed by hemoptysis. A repeat chest X-ray (Figure 3) showed a left-sided hydropneumothorax, for which an intercostal tube was inserted and the drainage was hemorrhagic. HRCT of the chest (Figure 4) revealed an encysted left-sided hydropneumothorax with aerated left upper lobe showing ground glass with cavitating consolidation. Peribronchovascular patchy consolidations with ground glassing were noted in the right lung with upper lobe predominance. BAL fungal culture later isolated *Aspergillus *species.

He was started on weight-based voriconazole (200 mg twice daily), but due to deranged liver biochemistry, voriconazole had to be switched to liposomal amphotericin 200 mg daily after three days. Despite optimal treatment, he deteriorated further and progressed to respiratory failure, requiring intubation and mechanical ventilation. Unfortunately, despite all attempts, he passed away on the second day of the intensive care unit (ICU).

Case 3

A 67-year-old man with multiple comorbidities, including hypertension, diabetes mellitus, chronic kidney disease, and ischemic heart disease for which he had undergone coronary artery bypass grafting (CABG), was transferred to our tertiary care center from a local hospital. He had been experiencing fever with chills and rigors for two weeks, along with a productive cough with rusty-colored sputum prior to the presentation.

On examination, he was febrile and ill-looking but hemodynamically stable, with a SpO₂ of 96%, blood pressure of 140/90 mmHg, and a pulse rate of 90 bpm. Lung auscultation revealed bilateral coarse crepitations with occasional rhonchi diffusely heard throughout both lung fields.

**Figure 5:**
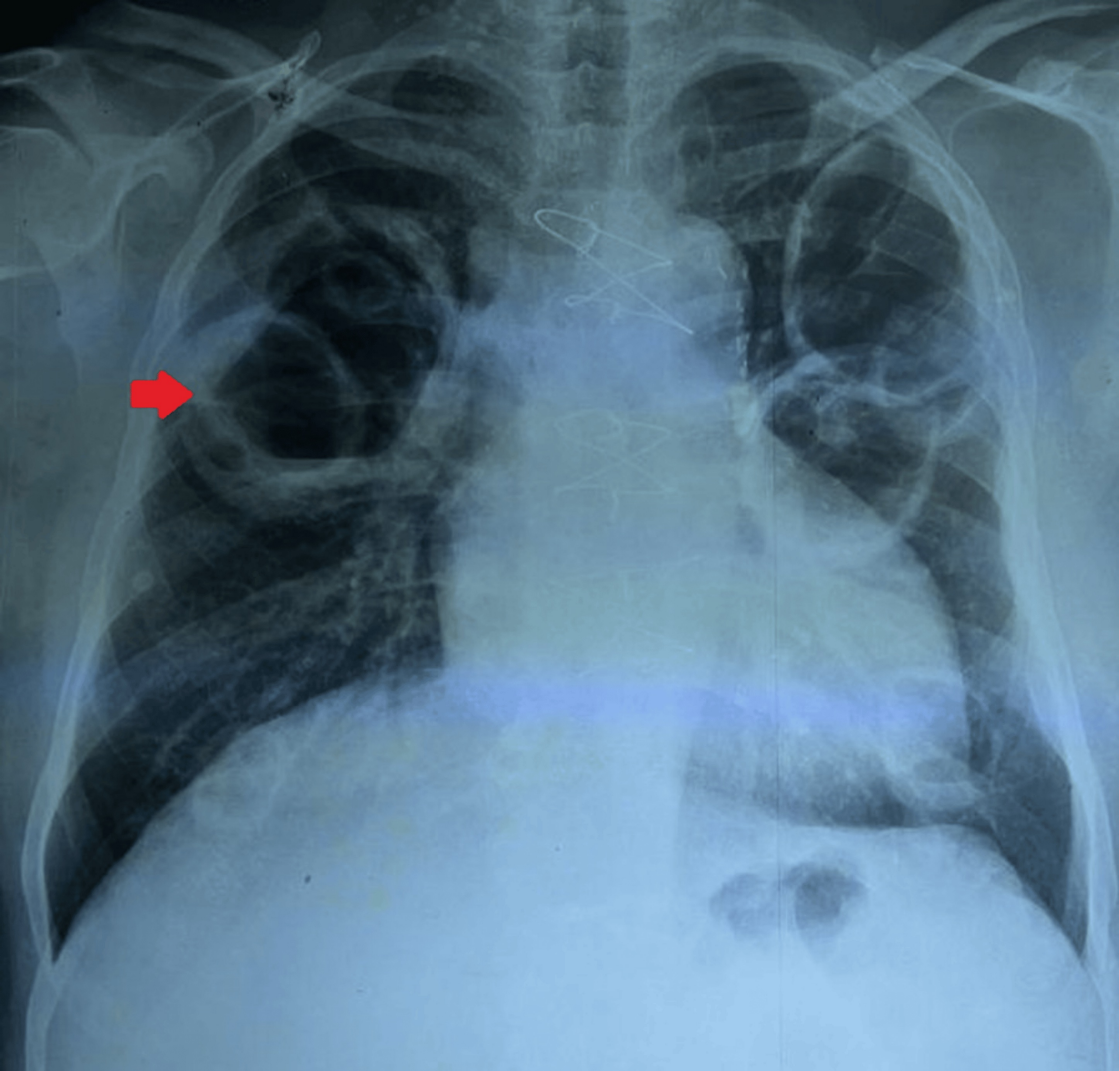
Chest radiograph (patient 3) Chest radiograph showing bilateral cavitary lesions (red arrow) predominantly involving the bilateral upper lobes

**Figure 6:**
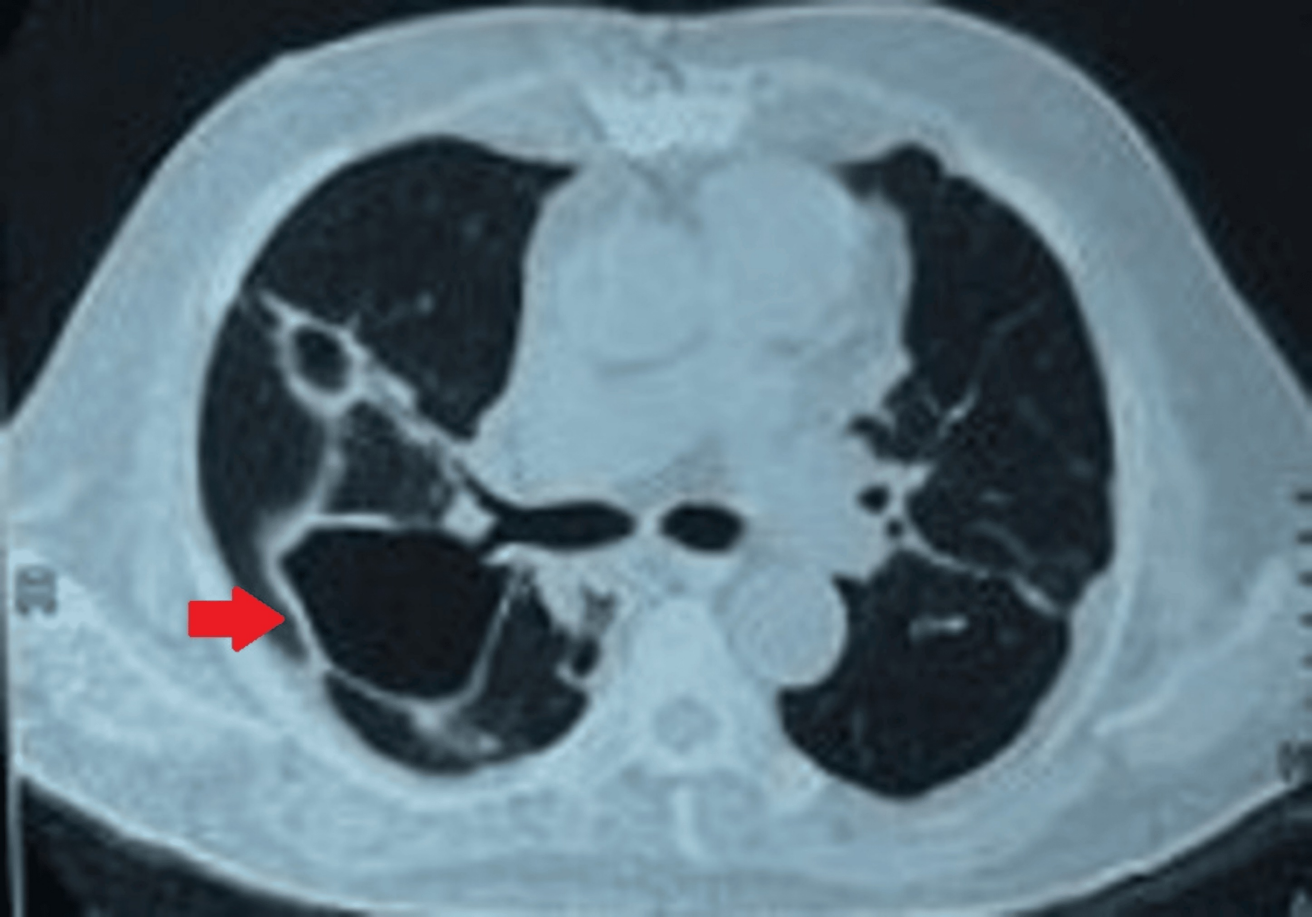
HRCT of the chest (patient 3) HRCT of the chest illustrating multiple bilateral cavitary lesions with internal air loculi (red arrow), associated with patchy ground-glass opacities and peribronchial thickening, suggestive of necrotizing pneumonia HRCT: high-resolution computed tomography

Laboratory investigations showed neutrophilic leukocytosis with markedly elevated inflammatory markers. Baseline renal and liver biochemistry was deranged. COVID-19 was reported as negative. The chest radiograph (Figure 5) revealed bilateral cavitating lesions. HRCT of the chest (Figure 6) demonstrated multiple bilateral cavitating lesions with internal air loculi, associated with patchy ground-glass opacities and peribronchial thickening, suggestive of necrotizing pneumonia.

Sputum pyogenic culture isolated* Candida albicans*, while the bronchial wash culture was positive for *Pseudomonas* species. Both sputum and bronchial wash samples were negative for tuberculosis. BAL galactomannan was positive (Table 1), and BAL fungal culture isolated *Aspergillus fumigatus*. Autoimmune screening, including antinuclear antibody (ANA) and c- and p-antineutrophil cytoplasmic antibodies (ANCA), was negative. Melioidosis screening was also negative. A two-dimensional echocardiogram (2D echo) did not demonstrate features suggestive of infective endocarditis. The ultrasound of the abdomen and pelvis revealed acute-on-chronic parenchymal changes in both kidneys, with no evidence of hepatosplenomegaly. Blood cultures were negative.

He was commenced on intravenous meropenem 1 g three times a day (tds) based on the antimicrobial sensitivity pattern. Intravenous voriconazole 6 mg/kg every 12 hours was started for the first 24 hours, followed by the maintenance dose of 4 mg/kg twice daily dose, which had been administered for nine days prior to transfer and had to be withheld due to worsening liver function. Following discussion with the microbiology team, intravenous liposomal amphotericin B (200 mg diluted in 500 mL of 5% dextrose) was initiated and administered over a 4-5-hour infusion. Treatment was continued for 10 days with close biochemical monitoring, including the daily assessment of serum creatinine, blood urea, sodium, and potassium, and the alternate-day monitoring of liver biochemistry, full blood count, serum calcium, and magnesium. The patient subsequently demonstrated both clinical and radiological improvement. Repeat fungal investigations performed 40 days after the initiation of antifungal therapy (BAL galactomannan 0.53 and serum aspergillus IgG) were negative. Follow-up HRCT showed significant interval resolution.

## Discussion

Invasive pulmonary aspergillosis accounts for high mortality and morbidity, especially in patients with underlying immunosuppression or multiple comorbidities. A retrospective hospital-based study done involving 216 patients with invasive pulmonary aspergillosis revealed that the overall mortality could be as high as 68.5% [4]. Another retrospective cohort study done in patients with acute-on-chronic liver failure with IPA showed all-cause mortality approaching nearly 95% [5].

This case series describes the types of presentation and the challenges in management, as well as the outcomes associated with invasive pulmonary aspergillosis. Radiological features include nodularity with or without cavitation, peribronchial infiltrates with or without tree-in-bud pattern or patchy or segmental consolidation [6]. The second case describes the presence of hydropneumothorax in addition to the conventional radiological findings. According to the literature, hydropneumothorax is a rare presentation of IPA, and the mechanisms underlying its formation remain poorly understood [7].

The diagnosis of IPA can be categorized as noninvasive and invasive modalities. Serum biomarkers such as galactomannan, beta-D-glucan assay, polymerase chain reaction (PCR), and sputum for fungal staining and culture are considered noninvasive, whereas bronchoscopy with bronchoalveolar lavage, transbronchial biopsy, computed tomography (CT)-guided transthoracic needle biopsy, and video-assisted thoracoscopic surgery (VATS) are recognized as invasive methods of diagnosis [8]. In all three patients, *Aspergillus* species were isolated from BAL fungal cultures, and in Cases 1 and 3, a positive serum galactomannan served as a supportive surrogate marker.

Management includes antifungal treatment, with voriconazole being the first line, which had been withheld in all three cases due to the development of drug-induced hepatitis necessitating the use of liposomal amphotericin. Posaconazole or isavuconazole are preferred options, available in both oral and intravenous formulations, whereas amphotericin is only available as an intravenous preparation and is used when voriconazole is not tolerated [9]. The adverse effect profile must be closely monitored during treatment. Voriconazole is particularly associated with liver dysfunction, visual disturbances, and neurotoxicity, while amphotericin is associated with electrolyte imbalances due to proximal renal tubular acidosis and with nephrotoxicity.

The duration of antifungal therapy is based on the site of the infection, the patient's immune status, and clinical and radiological response to treatment. The minimal duration of therapy is 6-12 weeks, but for immunosuppressed individuals, the duration may have to be extended from months to years [9]. This describes the need for close surveillance while on antifungal treatment and the need for individualized treatment strategies for optimal management. Cases 1 and 3 illustrate the successful treatment of IPA with favorable clinical, biochemical, and radiological outcomes. Case 2 describes the high mortality risk associated with immunosuppression, particularly due to poorly controlled diabetes mellitus and when complicated with hydropneumothorax and respiratory decline.

This case series also highlights the role of concomitant infections associated with IPA, as seen in Case 3, where both *Pseudomonas* species and *Candida albicans* were isolated from bronchial wash and sputum samples, illustrating polymicrobial involvement that can further aggravate disease severity.

## Conclusions

Invasive pulmonary aspergillosis poses significant diagnostic and therapeutic challenges, particularly in patients with immunosuppression and multiple comorbidities. The management of IPA is highly individualized, as voriconazole, which remains the first-line treatment, is not always applicable due to adverse drug reactions or the risk of target organ involvement. In such cases, alternatives such as liposomal amphotericin, posaconazole, or isavuconazole may be necessary to overcome therapeutic challenges.

The three cases presented here illustrate the broad clinical and radiological spectrum of IPA, notably highlighting the rare manifestation of hydropneumothorax observed in patient 2, underscoring the atypical and sinister nature of pulmonary complications. These cases emphasize the validity of maintaining a high degree of suspicion for IPA, especially in high-risk populations, and adopting a multidisciplinary approach involving respiratory physicians, radiologists, microbiologists, mycologists, and intensive care teams for early detection, microbiological evaluation, and the initiation of patient-specific therapy to improve clinical outcomes.
